# Self-efficacy and implementation intentions in home rehabilitation of stroke patients: the parallel mediating role of recurrence risk perception and outcome expectations

**DOI:** 10.3389/fpsyg.2025.1656514

**Published:** 2025-09-02

**Authors:** Xiaowen Jiang, Qiuxue Sun, Rong Tang, Shuxian Liu, Xi Chen, Yumei Lv

**Affiliations:** Department of Nursing, Harbin Medical University, Harbin, China

**Keywords:** stroke, home-based rehabilitation, implementation intentions, self-efficacy, recurrence risk perception, outcome expectations

## Abstract

**Background:**

Self-efficacy can improve the implementation intentions level of rehabilitation exercise in stroke patients. Yet, the underlying mechanism of benefits remains unclear especially in the home-based environment.

**Objective:**

This study aims to assess the level of implementation intentions in home-based rehabilitation exercises among stroke patients, clarify the relationship between self-efficacy and implementation intention, and determine whether recurrence risk perception and outcome expectations mediate this relationship.

**Methods:**

We conducted a quantitative cross-sectional study, recruiting 216 stroke patients who met the inclusion and exclusion criteria from three communities in Daqing City between June 2024 and April 2025. The mediating effects of recurrence risk perception and outcome expectations were assessed using Model 4 (parallel mediation) of the SPSS PROCESS macro with the bootstrap method.

**Results:**

The results showed that the score of home rehabilitation exercise implementation intentions of stroke patients was 60.62 ± 6.87, which still needs to be improved. Mediation analysis showed that self-efficacy played a significant direct role in executive intention, and recurrence risk perception and outcome expectations mediated the relationship between self-efficacy and the implementation intentions.

**Conclusion:**

There is considerable room for improvement in the implementation intentions of home rehabilitation exercise in stroke patients. This can be enhanced by intervening in rehabilitation self-efficacy, which in turn can influence recurrence risk perception and outcome expectations to improve the level of implementation intentions.

**Impact:**

This study aims to draw the attention of healthcare providers and family members to patients’ self-efficacy, recurrence risk perception, and outcome expectations, and advocate that the above variables can be used as the focus of future intervention in patients ‘home rehabilitation exercise implementation intentions.

## 1 Introduction

The American Heart Association/American Stroke Association defines stroke as an acute central nervous system (CNS) injury resulting from vascular causes, including cell death in the brain, spinal cord, or retina, accompanied by neurological deficits, manifested as symptoms such as facial asymmetry, limb weakness, speech disorders (aphasia/dysarthria), and ataxia (Sacco et al., 2013). From an etiological perspective, approximately 85% of strokes are ischemic, caused by blocked cerebral arteries due to thromboembolism (Dohle et al., 2025). According to the latest Global Burden of Disease (GBD) report, stroke has become the second leading cause of global mortality and the third leading cause of death and disability among non-communicable diseases (NCD) (Thayabaranathan et al., 2022). With evolving lifestyles, the global incidence of stroke has generally been on the rise since 1990 to 2021 (Feigin et al., 2024). In addition, the WSO/Lancet Committee on Stroke Neurology predicts that the number of stroke deaths worldwide will increase by 50% from 2020 to 2050 (Feigin and Owolabi, 2023). A study showed that 75% of stroke patients are accompanied by varying degrees of physical impairment, and rehabilitation exercise is their preferred rehabilitation treatment, but their compliance level is generally low, especially home rehabilitation exercise compliance (Bukhari et al., 2023; García-Cabo and López-Cancio, 2020). Yao et al. (2017) showed that during the 6 ~ 21 weeks home recovery period, patients’ exercise compliance began to decline. In addition, the majority of home stroke patients had not yet reached the American Heart Association’s recommendation of 30 min of moderate to vigorous physical activity per day (Fini et al., 2017). This gap occurs because they remained in the “knowing but not doing” stage: they possess behavioral intentions for rehabilitation exercise but fail to translate these intentions into sustained exercise routines (Jieqiong et al., 2019).

Implementation intentions are mediating variables between behavioral intentions and behaviors, which can not only promote the conversion from intention to behavior but also facilitate the formation of habitual behaviors (Lijuan and Danheng, 2020). Implementation intentions are specific action plans that tell us when, where, and how to achieve our goals, with specific forms including action plans and coping plans. The core framework operates on the “If scenario X occurs, then I’ll perform action Y!” principle (Schwarzer, 2008). The action plan specifies concrete contexts for executing target behaviors. For example: If it’s 9 AM on a workday, I’ll complete 30 min of upper body training in the living room. The coping plan anticipates alternative strategies for common obstacles. For instance: If walking training causes increased pain, I’ll immediately stop and switch to seated ankle pump exercises. Forming implementation intentions not only mitigates detrimental habits (e.g., reducing smoking frequency and alcohol consumption) (Feigin and Owolabi, 2023; Cooke et al., 2023), but also facilitates the adoption of health-promoting behaviors. Forming exercise-related implementation intentions can improve the activity ability of patients with multiple sclerosis while reducing their fatigue (Malaguti et al., 2020). A meta-analysis showed that implementation intentions effectively promote rehabilitation exercise behaviors (e.g., increased exercise duration) and improve dietary compliance among community-dwelling chronic disease patients (Lin et al., 2022). However, the level of implementation intentions for rehabilitation exercise in stroke patients still needs improvement (Ru et al., 2023). Improving the implementation intentions level of rehabilitation exercises in stroke patients potentially may enhance exercise adherence, potentially contributing to improved quality of life, though these potential effects require further validation. Therefore, identifying determinants of implementation intentions and exploring the potential mechanisms of these factors are of great significance.

Rehabilitation self-efficacy refers to the confidence level of stroke patients in completing rehabilitation exercises or daily activities. Multiple studies have confirmed the correlation between self-efficacy and implementation intentions, with higher self-efficacy predicting greater fidelity to planned actions (Shirui et al., 2024; Churchill et al., 2019). Shanshan et al. (2021a) showed a significant positive correlation between rehabilitation self-efficacy and implementation intentions for rehabilitation exercise in stroke patients, indicating that self-efficacy levels directly correspond to implementation intention strength. Conversely, patients with lower self-efficacy frequently exhibit poor rehabilitation exercise behaviors due to a lack of confidence in overcoming difficulties and poor self-regulation abilities when facing challenges.

Outcome expectations refer to an individual’s belief-based subjective cognitions regarding anticipated behavioral consequences, serving as a critical determinant for initiating and maintaining exercise regimens (Bandura, 1997). According to Bandura’s self-efficacy theory, an individual’s sense of self-efficacy is the core cognitive basis of outcome expectations (Bandura, 1977). Empirical evidence confirms significant correlations between self-efficacy and exercise-related outcome expectations, patients with lower self-efficacy develop more negative outcome beliefs, thereby compromising their outcome expectations (Chu and Wang, 2022). Furthermore, research in kidney transplant recipients demonstrates that stronger positive outcome expectations predict more detailed exercise planning and higher implementation intention levels, consequently promoting rehabilitation exercise adherence (Yue, 2020). Given the relationship between outcome expectations, self-efficacy, and implementation intentions, we hypothesize that outcome expectations play a mediating role in the relationship between the two.

Recurrence risk perception refers to the awareness of warning characteristics related to the severity of recurrence, behavior-related risk factors, and disease-related risk factors (Beilei et al., 2021). According to Rogers’ protection motivation theory, self-efficacy modifies risk perception by reconstructing threat assessment parameters (severity and susceptibility) (Maddux and Rogers, 2025). A recent study (Jingjing et al., 2024) shows that self-efficacy can not only directly affect compliance with rehabilitation exercises but also indirectly influence compliance through disease perception. Recurrence risk perception has a positive impact on the health behaviors of stroke patients. Patients with higher levels of recurrence risk perception exhibit stronger rehabilitation confidence and proactively pursue exercise strategies, resulting in heightened implementation intentions (Wang et al., 2024; Yao and Xiaofang, 2022). However, some studies have shown that compared to other influencing factors, the impact of risk perception is weaker and not significant (Lippke et al., 2004). Therefore, this study will further verify the relationship between recurrence risk perception and implementation intentions.

The Health Action Process Approach (HAPA), proposed by Schwarzer (2008), is a new stage-based health behavior theory. It holds that the generation and maintenance of health behaviors are a continuous and dynamic process, and factors such as risk perception, outcome expectations, self-efficacy and other factors can affect implementation intentions. Although previous studies have confirmed the impact of rehabilitation self-efficacy on implementation intentions, its mediating pathways remain underexplored. Therefore, this study will explore the relationships among self-efficacy, implementation intentions, recurrence risk perception, and outcome expectations in stroke populations. Based on theoretical guidance and literature review, this study hypothesizes that rehabilitation self-efficacy influences implementation intentions both directly and indirectly through two parallel mediators: recurrence risk perception and outcome expectations.

## 2 Methods

### 2.1 Study design and participants

Based on Kendall’s sample size calculation formula (Weber and Kendall, 1977): the sample size should be 5 to 10 times the number of variables. This study has 27 variables, considering 20% invalid samples. A total of 216 stroke patients from 3 communities in Daqing City, Heilongjiang Province, China.

Who met the inclusion and exclusion criteria from June 2024 to April 2025 were selected. Inclusion criteria: (1) Diagnosed according to the diagnostic criteria in the “Diagnostic criteria of cerebrovascular diseases in China (version 2019) “published by the Cerebrovascular Disease Group of the Neurology Branch of the Chinese Medical Association (Chinese Medical Association neurology branch, Chinese Medical Association neurology branch cerebrovascular disease group, 2019), confirmed by CT or MRI as stroke patients; (2) Age ≥ 18 years; (3) Conscious and stable after treatment, with certain communication, comprehension, and language abilities (as assessed by research nurses or community health providers); (4) Voluntarily participated in this study and signed the informed consent form. Exclusion criteria: (1) History of mental illness or obvious cognitive dysfunction (through review of community e-health records and self-reports by patients or their caregivers); (2) Combined organ failure of heart, liver, lung, or malignant tumors; (3) Currently participating in other studies. This study utilized convenience sampling. Our participants were family stroke survivors residing in specific communities during the study period (not continuous patients). The Recruitment Process: community nurses identified potentially eligible stroke survivors who met inclusion criteria. Researchers subsequently contacted these potential participants (or their primary caregivers), explained the study details, screened for fully qualified individuals, and obtained informed consent.

### 2.2 Ethical considerations

The study strictly followed the principles of voluntariness, confidentiality, and non-harmfulness. The informed consent was obtained from the study participants before the survey. Approval was obtained from the Ethics Review Committee of Daqing Campus, Harbin Medical University (Ethics No. 2022-R-164) on September 23, 2022.

### 2.3 Data collection procedures

Firstly, introduce the purpose and significance of the survey to the research participants and patients were assisted in signing the informed consent form. Members of the team collected the filled questionnaires on site and checked them. A total of 220 questionnaires were distributed and 216 valid questionnaires were collected, with an effective recovery rate of 98.18%.

### 2.4 Research tools

#### 2.4.1 General information questionnaire

The questionnaire was designed by the researcher based on a literature review and mainly includes information such as gender, age, educational level, marital status, average monthly income, medical expenses, number of disease occurrences, type of stroke, and number of comorbidities.

#### 2.4.2 The stroke self-efficacy questionnaire

The scale was developed by Jones et al. (2008) specifically for stroke rehabilitation patients, and translated and debugged into Chinese by Hongyan et al. (2015). The scale includes 2 dimensions of daily life activity efficacy and self-management efficacy, 11 items, and is scored on a 0–10 scale with a total score of 0–110, with higher scores representing higher rehabilitation self-efficacy. Its Cronbach’s *α* coefficient is 0.965, which has good reliability and validity.

#### 2.4.3 Implementation intention questionnaire for stroke patients’ rehabilitation exercise behaviors

This scale was developed by Shanshan et al. (2021b). The questionnaire includes two dimensions: action planning (8 items) and coping planning (11 items), with a total of 19 items. The items use a 5-point Likert scale, scored from 1 to 5 (ranging from “completely disagree” to “completely agree”), with a total score ranging from 19 to 95. Higher scores indicate a stronger intention to perform rehabilitation exercise behaviors. Implementation intention levels were categorized based on the composite score index, calculated as: Score Index = (Actual Score / Maximum Possible Score) × 100%. <60% indicated suboptimal implementation intention, 60% ~ 80% indicated moderate implementation intention, and >80% indicated strong implementation intention (Shanshan et al., 2021a). The content validity index of the questionnaire is 0.947, Cronbach’s *α* coefficient is 0.980, and test–retest reliability is 0.880.

#### 2.4.4 Recurrence risk perception scale for patients with stroke

This scale was developed by Beilei et al. (2021) and consists of 2 parts. Part 1 assesses the perception of the likelihood of recurrence, including 3 items, with a total score ranging from 1 to 25. Part 2 includes the perception of the severity of recurrence (7 items), the perception of disease-related risk factors (4 items), and the perception of behavior-related risk factors (6 items), totaling 17 items across 3 dimensions; a 3-point scale is used: “Disagree,” “Uncertain,” and “Agree,” scored as 1, 2, and 3 points, respectively. The total score for both parts ranges from 18 to 76, with higher scores indicating a higher perception of the recurrence risk perception in stroke patients. The overall Cronbach’s *α* coefficient of the scale is 0.850, and the Cronbach’s α coefficients for each dimension are 0.875, 0.815, and 0.804.

#### 2.4.5 Outcome expectation scale

The scale was developed by Schwarzer (2008), including two dimensions: positive outcome expectations, 10 items, and 3 items in the negative expectations, for a total of 13 items. Mengying (2011) translated and localized the scale into Chinese, with Cronbach’s *α* coefficients of 0.886 and 0.564 for positive and negative outcome expectancy, respectively. Outcome expectations levels were categorized based on the composite score index, calculated as: Score Index = (Actual Score / Maximum Possible Score) × 100%. <60% indicated suboptimal outcome expectations, 60% ~ 80% indicated moderate outcome expectations, and >80% indicated strong outcome expectations.

### 2.5 Data analysis

SPSS 27.0 was used for data analysis. Continuous variables with a normal distribution are expressed as (x ± s), count data are presented as cases (%), correlation analysis was conducted using Pearson correlation analysis; the mediation effect was tested using PROCESS 4 developed by Hayes (2013), and the significance of the mediation effect was tested using the Bootstrap method. *p* < 0.05 was considered statistically significant.

## 3 Results

### 3.1 General characteristics of participants

A total of 216 stroke patients were included in this study, and their demographic and clinical data are shown in Table 1. Among them, 141 (65.3%) were male and 75 (34.7%) were female; nearly half of the patients (48.1%) were ≥60 years of age, and most of them (81.5%) were married; most of the patients (77.4%) had received junior high school education or above, and 53.3% of the patients had a personal income of more than 3,000 yuan per month. In addition, more than half of the patients (57.9%) had their first disease, 77.3% had ischemic stroke, and 84.3% had two or more co-morbidities.

**Table 1:** General information of stroke patients (*N* = 216).

Variables	Categories	*N*	%
Gender	Male	141	65.3
	Female	75	34.7
Age	<60	112	51.9
	≥60	104	48.1
Place of residence	Rural	64	29.6
	Urban	152	70.4
Marital status	Married	176	81.5
	Single	40	18.5
	Middle school and below	134	62.0
	High school and above	82	38.0
	Employment	121	56.0
	Retired	68	31.5
	Unemployed	27	12.5
Average monthly income (RMB, yuan)	<3,000	101	46.7
	3,000–4,999	82	38.0
	≥5,000	33	15.3
Medical expenses	Rural Cooperative Medical Care	74	34.3
	Urban Insurance	53	24.5
	Employee insurance	86	39.8
	Other	3	1.4
	spouse and children	192	88.8
	Living alone	19	8.8
	Other	5	1.4
	spouse and children	199	92.1
	Other	17	7.9
Number of episodes	First episode	125	57.9
	Recurrence once	75	34.7
	Recurrence twice or more	16	7.4
Type of stroke	Hemorrhagic stroke	49	22.7
	Ischemic stroke	167	77.3
Number of chronic diseases	1	34	15.7
	2	119	55.1
	3 or more	63	29.2
Current course of disease	≤6 months	137	63.4
	>6 months	79	36.6
Rehabilitation history	Yes	52	24.1
	No	164	75.9
Assistive devices	Yes	52	24.1
	No	164	75.9
Self-Care Ability	Mostly dependent	12	5.6
	Partially dependent	53	24.5
	Slightly dependent	145	67.1
	Independently completed	6	2.8

### 3.2 Common method bias test

The Harman single-factor test was used to examine common method bias in the samples, extracting a total of 17 factors with eigenvalues greater than 1. The maximum factor variance explanation rate was 22.9%, significantly below the critical threshold of 40%. Therefore, there is no serious common method bias in this study.

### 3.3 Rehabilitation self-efficacy, implementation intentions, recurrence risk perception, and outcome expectation scale scores of stroke patients

The score of home rehabilitation exercise implementation intention was 60.62 ± 6.87, the score of action plan dimension was 24.74 ± 3.28, and the score of coping plan dimension was 35.89 ± 4.24. The score of rehabilitation self-efficacy was 64.45 ± 7.04, the score of daily living activities efficacy dimension was 34.50 ± 5.18, the score of self-management efficacy dimension was 29.95 ± 3.98, the score of recurrence risk perception was 48.33 ± 6.05, the score of perception of severity of recurrence dimension was 18.09 ± 1.95, the score of perception of disease-related risk factors dimension was 9.03 ± 1.71, and the score of perception of behavior-related risk factors dimension was 15.59 ± 1.88. The score of outcome expectations was 42.59 ± 4.52, the score of positive outcome expectations dimension was 32.80 ± 3.31, and the score of negative outcome expectations dimension was 9.80 ± 2.38 (as shown in Table 2). The mean entry score showed that the coping plan score (3.26 ± 0.39) was significantly higher than the action plan score (3.09 ± 0.41), *t* = −7.578, *p* < 0.001, *d* = 0.33, 95% CI [−0.22, −0.13].

**Table 2:** Scores of self-efficacy, recurrence risk perception, outcome expectations, and rehabilitation exercise implementation intentions scores in stroke patients (*N* = 216).

Variable	Dimension	Actual score range	Mean ± standard deviation	Mean entry score	Scoring rate
Implementation intention		19–95	60.62 ± 6.87	3.19 ± 0.36	63.81%
	Action plan	8–40	24.74 ± 3.28	3.09 ± 0.41	61.85%
	Coping plan	11–55	35.89 ± 4.24	3.26 ± 0.39	65.25%
Self-efficacy		0–110	64.45 ± 7.04	5.86 ± 0.64	58.59%
	Daily life activity efficacy	0–60	34.50 ± 5.18	5.75 ± 0.86	57.50%
	Self-management efficacy	0–50	29.95 ± 3.98	5.99 ± 0.80	59.90%
Recurrence risk perception		18–76	48.33 ± 6.05	2.42 ± 0.30	63.59%
	Perception of the severity of recurrence	7–21	18.09 ± 1.95	2.58 ± 0.28	86.14%
	Perception of disease-related risk factors	4–12	9.03 ± 1.71	2.26 ± 0.43	75.25%
	Perception of behavior-related risk factors	6–18	15.59 ± 1.88	2.60 ± 0.31	86.61%
Outcome expectations		13–65	42.59 ± 4.52	3.28 ± 0.35	65.52%
	Positive outcome expectation	10–50	32.80 ± 3.31	3.28 ± 0.33	65.60%
	Negative outcome expectation	3–15	9.80 ± 2.38	3.27 ± 0.79	65.33%

### 3.4 Correlation analysis of self-efficacy, implementation intentions, recurrence risk perception, and outcome expectations in stroke patient rehabilitation

Correlation analysis was conducted on the total scores of four variables: rehabilitation self-efficacy, implementation intention, recurrence risk perception, and outcome expectation. The results showed that implementation intention was positively correlated with rehabilitation self-efficacy (*r* = 0.409, *p* < 0.001), recurrence risk perception (*r* = 0.439, *p* < 0.001), and outcome expectation (*r* = 0.562, *p* < 0.001), as shown in Table 3.

**Table 3:** Correlations between the main study variables (*N* = 216).

Variable	Self-efficacy	Implementation intention	Recurrence risk perception	Outcome expectations
Self-efficacy	1	—	—	—
Implementation intention	0.409**	1	—	—
Recurrence risk perception	0.412**	0.439**	1	—
Outcome expectations	0.434**	0.562**	0.434**	1

^**^p < 0.01.

### 3.5 Parallel mediation effect analysis

**Figure 1:**
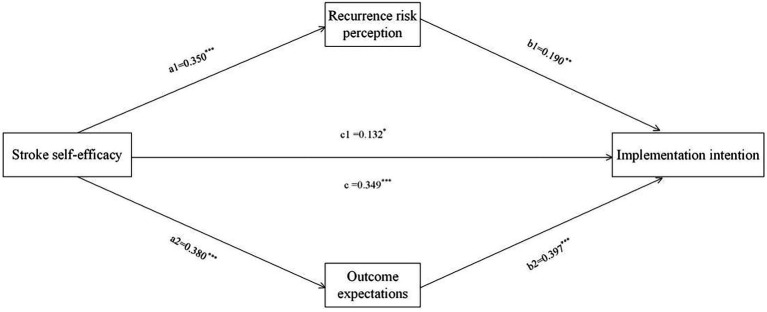
A mediating model of recurrence risk perception and outcome expectation between self-efficacy and implementation intention of rehabilitation exercises (*N* = 216). The coefficient c is the total effect between self-efficacy and the intention to perform rehabilitation exercises, and c1 is the direct effect of self-efficacy on the intention to perform rehabilitation exercises. *p<0.05, **p<0.01, ***p<0.001.

Previous studies have shown that gender, age, literacy, personal monthly income, number of co-morbidities, and number of episodes affect the patients’ implementation intentions, so they were used as control variables. The analysis was conducted with the total score of implementation intentions as the outcome variable, the total score of rehabilitation self-efficacy as the predictor variable, and the total score of recurrence risk perception and the total score of outcome expectations as the mediator variables. PROCESS (Model 4) was used, with the Bootstrap method resampling 5,000 times, and a 95% confidence interval set for testing the mediating effect. The results of the mediated effects analysis are shown in Figure 1 and Table 4. The results showed that the direct (path c1 = 0.132, 95% CI = 0.007, 0.257) and total (path c = 0.349, 95% CI = 0.224, 0.475) effects of rehabilitation self-efficacy on implementation intentions were significant. In addition, recurrence risk perception (path a1b1 = 0.067, 95% CI = 0.021, 0.114) and outcome expectations (path a2b2 = 0.151, 95% CI = 0.083, 0.236) partially mediated the relationship between rehabilitation self-efficacy and implementation intentions, accounting for 19.19 and 43.26% of the total effect of rehabilitation self-efficacy on implementation intentions, respectively. None of the confidence intervals in the model test for this study contained 0, indicating that the paths all reached the level of significance.

**Table 4:** Mediation analysis of self-efficacy and Implementation intention of rehabilitation exercise (*N* = 216).

Variable	Effect	SE	LLCI	ULCI
Total effect	0.349	0.064	0.224	0.475
Direct effect	0.132	0.063	0.007	0.257
Total indirect effect	0.217	0.045	0.132	0.310
Self-efficacy → Recurrence risk perception → Implementation intention	0.067	0.024	0.021	0.114
Self-efficacy → Outcome expectations → Implementation intention	0.151	0.039	0.083	0.236

## 4 Discussion

Our study verified the impact of rehabilitation self-efficacy on the implementation intention of home rehabilitation exercises in stroke patients, indicating that rehabilitation self-efficacy helps improve patients’ implementation intentions. Additionally, this study demonstrated the parallel mediation of recurrence risk perception and outcome expectations in the relationship between rehabilitation self-efficacy and implementation intention. This study has several advantages. Firstly, it is the first study to identify recurrence risk perception and outcome expectations as parallel mediators between rehabilitation self-efficacy and implementation intentions. Secondly, our results reveal a possible relationship between self-efficacy and implementation intentions for stroke patients. Therefore, combining community rehabilitation with interventions targeting rehabilitation self-efficacy, recurrence risk perception, and outcome expectations can improve stroke patients’ implementation intentions for home rehabilitation exercises, thereby potentially enhancing exercise adherence, which may subsequently contribute to improved quality of life, though these potential effects require further validation. Additionally, considering factors such as rehabilitation self-efficacy, recurrence risk perception, and outcome expectations when formulating implementation intention plans may be an effective exercise strategy (Park and Kang, 2024).

The post-stroke rehabilitation period requires sustained management, and long-term hospitalization for recovery is not practical. Returning to community and home-based rehabilitation is often the preferred choice and has been proven to improve the quality of life for stroke patients (Ali et al., 2021). However, 30–50% of patients discontinue rehabilitation exercise within the first year post-discharge (Levy et al., 2019). This gap occurs because patients develop behavioral intentions for rehabilitation exercises but fail to translate them into implementation intentions, ultimately undermining consistent exercise adherence. Lin et al. (2022) research shows that implementation intentions can effectively improve the physical activity levels of community chronic disease patients. The results of this study show that the implementation intention score was 60.62 ± 6.87, and the score rate was 63.81%, similar to the results of Song et al. (2023) (59.82 ± 16.67), which was in the middle level, indicating that the level of implementation intention for home rehabilitation exercise in stroke patients needs to be improved (Shanshan et al., 2021a). The results of this study show that the coping plan score rate is higher than the action plan score rate, indicating that patients effectively identify exercise-related abnormalities and proactively seek clinical assistance, reflecting strong problem-solving efficacy. Conversely, the low action plan score rate indicates that patients have poor willingness for rehabilitation exercise, which may be related to insufficient knowledge about home rehabilitation exercise, leading to a lack of ability to formulate their own rehabilitation plans and difficulty putting them into action (Ru et al., 2023). Therefore, it is necessary to enhance patients’ cognitive level of home rehabilitation exercise, improve their ability to formulate rehabilitation exercise plans, and encourage patients to actively communicate with medical staff when encountering difficulties and obstacles, promoting the formation of implementation intentions for rehabilitation exercise behavior, thereby enabling patients to develop regular rehabilitation exercise behavior.

After analysis, our study found that rehabilitation self-efficacy has a direct effect on the implementation intentions of home rehabilitation exercises in stroke patients, which is consistent with the findings of Churchill et al. (2019). Individuals with high self-efficacy are more likely to act according to their implementation intentions than those with low self-efficacy, possibly because individuals with high self-efficacy set higher goals and have more effective solutions when encountering difficulties (Szczepańska-Gieracha and Mazurek, 2020). Conversely, individuals with low self-efficacy are prone to negative emotions such as depression and anxiety, which hinder exercise behavior (Kandola et al., 2019). In addition, stroke patients with high self-efficacy usually have more confidence in participating in daily life activities, are more capable of overcoming difficulties and obstacles during the rehabilitation exercise process, and generally have better psychosocial functioning and well-being (Warner and Schwarzer, 2020). A study on patients with multiple sclerosis showed that self-efficacy is an important predictor of patients’ adherence to home exercise programs, and high levels of self-efficacy help improve patients’ compliance with home exercise programs and quality of life (Almarwani and Alosaimi, 2023). Therefore, the promoting effect of rehabilitation self-efficacy on implementation intentions of home rehabilitation exercises in stroke patients should be emphasized, actively providing patients with support and encouragement, helping them build confidence in rehabilitation, and improving their self-efficacy levels, so as to improve the level of patients’ implementation intentions of home rehabilitation exercises.

In our study, the recurrence risk perception score was 48.33 ± 6.05, lower than the findings of Yao and Xiaofang (2022). It may be due to the fact that most participants were first-time stroke patients with mild functional impairment and insufficient disease-specific knowledge, and had not yet realized the severity of recurrence. The outcome expectation score in this study was 42.59 ± 4.52, and the score rate was 63.81%, which was in the middle level, indicating that the level of outcome expectation still needs to be improved (Zhihui et al., 2021). Yao and Xiaofang (2022) showed a positive correlation between recurrence risk perception and implementation intentions of rehabilitation exercises in stroke patients, which is consistent with the results of this study. The results of a qualitative study showed that stroke patients with low levels of rehabilitation outcome expectations had weaker confidence in the recovery of their neurological deficits and were prone to avoidance behaviors during early rehabilitation (Min et al., 2023). Our mediation analysis demonstrates that elevated rehabilitation self-efficacy enhances recurrence risk perception and outcome expectations, thereby increasing implementation intentions for home-based rehabilitation exercises. The reason for this may be that patients with higher levels of self-efficacy tend to take the initiative to acquire rehabilitation knowledge and skills, and have a correct recurrence risk perception (Jingjing et al., 2024), and at the same time, such patients have a greater ability to perceive benefits, have positive outcome expectations, and develop exercise autonomy, which facilitates setting rehabilitation goals and firmly implementing them (Gothe, 2018; Gangwani et al., 2022). Therefore, in addition to emphasizing the direct effect of self-efficacy on the implementation intention of rehabilitation exercise, our study also demonstrated that rehabilitation self-efficacy can influence the implementation intention of rehabilitation exercises through recurrence risk perception and outcome expectations, suggesting that healthcare professionals should pay attention to the education of patients’ knowledge about the disease, assist them to establish a correct recurrence risk perception, and encourage them to establish confidence in adhering to long-term rehabilitation exercises, and respond to difficulties and obstacles in the rehabilitation process with a positive attitude, which may have positive significance for improving the implementation intention of home rehabilitation exercises in stroke patients.

This study has several limitations. Firstly, it is based on a cross-sectional design, so causal relationships cannot be established. Further research using a prospective longitudinal design is needed to obtain more accurate results. Secondly, conducting convenience sampling at community health service centers instead of using random or continuous sampling may introduce selection bias. Moreover, since the participants were exclusively from Daqing City’s communities, the relatively small sample size limits the generalizability of our findings. Future studies requiring larger and more representative samples are needed to validate these results. Thirdly, our study relied on structured clinical judgment and self/caregiver report, rather than standardized neuropsychological tests to assess cognitive function, communication capacity, and exclude psychiatric disorders. We cannot definitively rule out the presence of mild cognitive impairment, undiagnosed depression or anxiety, or subtle communication deficits in our sample. Future studies should incorporate brief, validated screening tools during participant recruitment to better control for these covariates and enhance sample characterization.

## 5 Conclusion

Our study shows that the level of implementation intentions of home rehabilitation exercises in stroke patients needs to be improved, and demonstrates that rehabilitation self-efficacy can directly and indirectly affect the implementation intentions of home rehabilitation exercises through recurrence risk perception and outcome expectations. The results suggest that enhancing self-efficacy may help increase patients’ recurrence risk perception and positive outcome expectations, thereby improving their implementation intentions of home rehabilitation exercises.

## Data Availability

The original contributions presented in the study are included in the article/supplementary material, further inquiries can be directed to the corresponding author/s.

## References

[ref1] Ali A. Tabassum D. Baig S. S. Moyle B. Redgrave J. Nichols S.. (2021). Effect of exercise interventions on health-related quality of life after stroke and transient ischemic attack: A systematic review and Meta-analysis. Stroke 52, 2445–2455. doi: 10.1161/STROKEAHA.120.032979, PMID: 34039033

[ref2] Almarwani M. Alosaimi B. (2023). Exercise self-efficacy and fatigue as predictors of adherence to home-based exercise among patients with multiple sclerosis. Patient Prefer. Adherence 17, 1441–1449. doi: 10.2147/PPA.S414884, PMID: 37342492 PMC10278867

[ref3] Bandura A. (1977). Self-efficacy: toward a unifying theory of behavioral change. Psychol. Rev. 84, 191–215. 847061 10.1037//0033-295x.84.2.191

[ref4] Bandura A. (1997). Self-efficacy: The exercise of control, vol. ix. New York, NY, US: W H Freeman/Times Books/ Henry Holt & Co, 604.

[ref5] Beilei L. Zhenxiang Z. Yunfei G.. (2021). Development and psychometric test of recurrence risk perception scale for patients with stroke. Chin. J. Nurs. 56, 1666–1671. doi: 10.3761/j.issn.0254-1769.2021.11.011

[ref6] Bukhari S. Yaghi S. Bashir Z. (2023). Stroke in young adults. J. Clin. Med. 12:4999. doi: 10.3390/jcm12154999, PMID: 37568401 PMC10420127

[ref7] Chinese Medical Association neurology branch, Chinese Medical Association neurology branch cerebrovascular disease group (2019). Key points for diagnosis of various major cerebrovascular diseases in China 2019. Chin. J. Neurol. 52, 710–715. doi: 10.3760/cma.j.issn.1006-7876.2019.09.003

[ref8] Chu S. F. Wang H. H. (2022). Outcome expectations and older adults with knee osteoarthritis: their exercise outcome expectations in relation to perceived health, self-efficacy, and fear of falling. Health Care 11:57. doi: 10.3390/healthcare11010057 36611517 PMC9819286

[ref9] Churchill S. Pavey L. Sparks P. (2019). The impact of autonomy-framed and control-framed implementation intentions on snacking behaviour: the moderating effect of eating self-efficacy. Appl. Psychol. Health Well Being 11, 42–58. doi: 10.1111/aphw.12142, PMID: 30302915

[ref10] Cooke R. McEwan H. Norman P. (2023). The effect of forming implementation intentions on alcohol consumption: A systematic review and meta-analysis. Drug Alcohol Rev. 42, 68–80. doi: 10.1111/dar.13553, PMID: 36173203 PMC10087331

[ref11] Dohle E. Ashok A. H. Bhakta S. Induruwa I. Evans N. R. (2025). Thrombus composition in ischaemic stroke: histological and radiological evaluation, and implications for acute clinical management. J. Thromb. Thrombolysis 58, 355–369. doi: 10.1007/s11239-025-03074-6, PMID: 40117100 PMC12009245

[ref12] Feigin V. L. Abate M. D. Abate Y. H. Abd ElHafeez S. Abd-Allah F. Abdelalim A.. (2024). Global, regional, and national burden of stroke and its risk factors, 1990–2021: a systematic analysis for the global burden of disease study 2021. Lancet Neurol. 23, 973–1003. doi: 10.1016/S1474-4422(24)00369-7, PMID: 39304265 PMC12254192

[ref13] Feigin V. L. Owolabi M. O. (2023). Pragmatic solutions to reduce the global burden of stroke: a world stroke organization–lancet neurology commission. Lancet Neurol. 22, 1160–1206. doi: 10.1016/S1474-4422(23)00277-6, PMID: 37827183 PMC10715732

[ref14] Fini N. A. Holland A. E. Keating J. Simek J. Bernhardt J. (2017). How physically active are people following stroke? Systematic review and quantitative synthesis. Phys. Ther. 97, 707–717. doi: 10.1093/ptj/pzx038, PMID: 28444348

[ref15] Gangwani R. Cain A. Collins A. Cassidy J. M. (2022). Leveraging factors of self-efficacy and motivation to optimize stroke recovery. Front. Neurol. 13:823202. doi: 10.3389/fneur.2022.823202, PMID: 35280288 PMC8907401

[ref16] García-Cabo C. López-Cancio E. (2020). Exercise and stroke. Adv. Exp. Med. Biol. 1228, 195–203. doi: 10.1007/978-981-15-1792-1_13, PMID: 32342459

[ref17] Gothe N. P. (2018). Correlates of physical activity in urban african american adults and older adults: testing the social cognitive theory. Ann. Behav. Med. 52, 743–751. doi: 10.1093/abm/kax038, PMID: 30124762 PMC6128373

[ref18] Hayes A. F. (2013). Introduction to mediation, moderation, and conditional process analysis: A regression-based approach, vol. xvii. New York, NY, US: The Guilford Press, 507.

[ref19] Hongyan L. Liang F. Ruixue B.. (2015). Reliability and validity study of the Chinese version of the stroke rehabilitation self-efficacy scale. Chin. J. Nurs. 50, 790–794. doi: 10.3761/j.issn.0254-1769.2015.07.005

[ref20] Jieqiong Z. Pingping H. Xinping O.. (2019). A qualitative study on factors promoting health behaviors in functional exercise for stroke patients. General Nursing 17, 346–349. doi: 10.12104/j.issn.1674-4748.2019.03.037

[ref21] Jingjing Q. Lamei L. Wenting L.. (2024). Role of disease perception and treatment confidence in the compliance of self-efficacy rehabilitation of stroke patients. Chin. J. Rehabil. Med. 39, 401–405. doi: 10.3870/zgkf.2024.07.004

[ref22] Jones F. Partridge C. Reid F. (2008). The stroke self-efficacy questionnaire: measuring individual confidence in functional performance after stroke. J. Clin. Nurs. 17, 244–252. doi: 10.1111/j.1365-2702.2008.02333.x, PMID: 18578800

[ref23] Kandola A. Ashdown-Franks G. Hendrikse J. Sabiston C. M. Stubbs B. (2019). Physical activity and depression: towards understanding the antidepressant mechanisms of physical activity. Neurosci. Biobehav. Rev. 107, 525–539. doi: 10.1016/j.neubiorev.2019.09.040 31586447

[ref24] Levy T. Laver K. Killington M. Lannin N. Crotty M. (2019). A systematic review of measures of adherence to physical exercise recommendations in people with stroke. Clin. Rehabil. 33, 535–545. doi: 10.1177/0269215518811903, PMID: 30458647 PMC6416703

[ref25] Lijuan W. Danheng Z. (2020). Associations among habitual behavior, implementation intention and physical activity of adolescents: application of model of extended theory of planned behavior. J. Shanghai Univ. Sport 44, 22–32. doi: 10.16099/j.sus.2020.02.003

[ref26] Lin H. Yu P. Yang M. Wu D. Wang Z. An J.. (2022). Making specific plan improves physical activity and healthy eating for community-dwelling patients with chronic conditions: A systematic review and Meta-analysis. Front. Public Health 10:721223. doi: 10.3389/fpubh.2022.721223, PMID: 35664117 PMC9160833

[ref27] Lippke S. Ziegelmann J. P. Schwarzer R. (2004). Behavioral intentions and action plans promote physical exercise: A longitudinal study with orthopedic rehabilitation patients. J. Sport Exerc. Psychol. 26, 470–483. doi: 10.1123/jsep.26.3.470

[ref28] Maddux J. Rogers R. (2025). Protection motivation and self-efficacy: A revised theory of fear appeals and attitude change. J. Exp. Soc. Psychol. 19, 469–479. doi: 10.1016/0022-1031(83)90023-9

[ref29] Malaguti A. Ciocanel O. Sani F. Dillon J. F. Eriksen A. Power K. (2020). Effectiveness of the use of implementation intentions on reduction of substance use: A meta-analysis. Drug Alcohol Depend. 214:108120. doi: 10.1016/j.drugalcdep.2020.108120, PMID: 32622228

[ref30] Mengying S. (2011). Intervention strategies for exercise behavior in Chinese adults: Integration of TPB and HAPA models [D]. Beijing: Beijing Sport University.

[ref31] Min Z. Qing W. Huiling S.. (2023). Influencing factors of early activities in patients with acute ischemic stroke:a qualitative study. Chin. J. Nurs. 58, 2112–2118. doi: 10.3761/j.issn.0254-1769.2023.17.009

[ref32] Park K. H. Kang H. Y. (2024). The effects of a self-efficacy theory-based exercise program for patients undergoing with total knee arthroplasty. J. Korean Acad. Nurs. 54, 547–562. doi: 10.4040/jkan.24027, PMID: 39663619

[ref33] Ru Z. Yiping C. Panpan H.. (2023). Effect of implementation intentions strategy based on temporal self-regulation theory on implementation intentions of rehabilitation exercise behavior in young and middle-aged stroke patients. Nurs. Res. 37, 3368–3373. doi: 10.12102/j.issn.1009-6493.2023.18.024

[ref34] Sacco R. L. Kasner S. E. Broderick J. P. Caplan L. R. Connors J. J. Culebras A.. (2013). An updated definition of stroke for the 21st century: a statement for healthcare professionals from the American Heart Association/American Stroke Association. Stroke 44, 2064–2089. doi: 10.1161/STR.0b013e318296aeca, PMID: 23652265 PMC11078537

[ref35] Schwarzer R. (2008). Modeling health behavior change: how to predict and modify the adoption and maintenance of health behaviors. Appl. Psychol. 57, 1–29. doi: 10.1111/j.1464-0597.2007.00325.x

[ref36] Shanshan Z. Chengmei S. Li Y. Miao T. Fuguo Y. Wenwen L.. (2021a). Rehabilitation exercise behavior implementation intentions and its influencing factors among patients with first stroke. J. Nurs. Sci. 36, 12–16. doi: 10.3870/j.issn.1001-4152.2021.09.012

[ref37] Shanshan Z. Chengmei S. Li Y. Miao T. Fuguo Y. Wenwen L.. (2021b). Development and reliability and validity testing of the rehabilitation exercise behavior execution intention questionnaire for stroke patients. Chinese Nursing Manage. 21, 664–669. doi: 10.3969/j.issn.1672-1756.2021.05.007

[ref38] Shirui L. Zhenxiang Z. Wenna W. Jie Z. Zhixin Z. (2024). The influence of environmental factors on self-efficacy in the community stroke patients. Chinese. Gen. Pract. 27, 3535–3539. doi: 10.12114/j.issn.1007-9572.2023.0699

[ref39] Song L. Huan H. Hongyu L. Hanjing Z. Yanli Z. Yuetong L. (2023). The chain mediation effect of rehabilitation exercise behavioral intention and mental health literacy between perceived social support and health behavior in hospitalized stroke patients. Military Nursing 40, 34–38.

[ref40] Szczepańska-Gieracha J. Mazurek J. (2020). The role of self-efficacy in the recovery process of stroke survivors. Psychol. Res. Behav. Manag. 13, 897–906. doi: 10.2147/PRBM.S273009, PMID: 33177896 PMC7649225

[ref41] Thayabaranathan T. Kim J. Cadilhac D. A. Thrift A. G. Donnan G. A. Howard G.. (2022). Global stroke statistics 2022. Int. J. Stroke 17, 946–956. doi: 10.1177/17474930221123175, PMID: 35975986 PMC9980380

[ref42] Wang X. Zhang Z. X. Lin B. L. Jiang H. Wang W. Mei Y. X.. (2024). Mediation role of perceived social support between recurrence risk perception and health behaviour among patients with stroke in China: a cross-sectional study. BMJ Open 14:e079812. doi: 10.1136/bmjopen-2023-079812, PMID: 38355172 PMC10868314

[ref43] Warner L. M. Schwarzer R. (2020). “Self-efficacy and health” in The Wiley encyclopedia of Health Psychology. eds. Sweeny K. Robbins M. L. Cohen L. M.. 1st ed (Wiley), 605–613. doi: 10.1002/9781119057840.ch111

[ref44] Weber E. Kendall M. (1977). Multivariate analysis. Charles Griffin & co. LTD. London, High Wycombe 1975. 210 s., 9 Abb., 27 tab., 1 Anhang, £ 6,80. Biometrical J. 19:309.

[ref45] Yao M. Chen J. Jing J. Sheng H. Tan X. Jin J. (2017). Defining the rehabilitation adherence curve and adherence phases of stroke patients: an observational study. Patient Prefer. Adherence 11, 1435–1441. doi: 10.2147/PPA.S139854, PMID: 28860726 PMC5572952

[ref46] Yao C. Xiaofang S. (2022). Current status of stroke patients' perception of recurrence risk and its correlation with rehabilitation exercise and medication adherence. General Nursing 20, 4306–4308. doi: 10.12104/j.issn.1674-4748.2022.30.034

[ref47] Yue H. (2020). A study on the current status and influencing factors of exercise in kidney transplant recipients [D]. Beijing: Beijing University of Chinese Medicine.

[ref48] Zhihui Z. Weimin H. Meilin Y. Liwei Z. Lijin D. Yufeng H. (2021). Study on the relationship between rehabilitation training, exercise self-efficacy, exercise outcome expectation and quality of life in stroke inpatients. Fujian J. Traditional Chinese Med. 52, 13–16. doi: 10.3969/j.issn.1000-338X.2021.01.005

